# Outcome of Assisted Reproductive Technology (ART) and Subsequent Self-Reported Life Satisfaction

**DOI:** 10.1371/journal.pone.0112540

**Published:** 2014-11-13

**Authors:** Paula Kuivasaari-Pirinen, Heli Koivumaa-Honkanen, Maritta Hippeläinen, Kaisa Raatikainen, Seppo Heinonen

**Affiliations:** 1 Department of Obstetrics and Gynaecology, Kuopio University Hospital, Kuopio, Finland; 2 Institute of Clinical Medicine, University of Eastern Finland, Kuopio, Finland; 3 Clinic of Child Psychiatry, Oulu University Hospital, Oulu, Finland; 4 Department of Psychiatry, Kuopio University Hospital, Kuopio, Finland; 5 Department of Psychiatry, South-Savonia Hospital District, Mikkeli, Finland; 6 Department of Psychiatry, North Karelia Central Hospital, Joensuu, Finland; 7 Department of Psychiatry, SOSTERI, Savonlinna, Finland; 8 Department of Psychiatry, SOTE, Iisalmi, Finland; 9 Department of Psychiatry, Lapland Hospital District, Rovaniemi, Finland; 10 Department of Psychiatry, Institute of Clinical Medicine, University of Oulu, Oulu, Finland; University of Pennsylvania, United States of America

## Abstract

**Objective:**

To compare life satisfaction between women with successful or unsuccessful outcome after assisted reproductive treatment (ART) by taking into account the time since the last ART.

**Design:**

Cohort study.

**Setting:**

Tertiary hospital.

**Patients:**

A total of 987 consecutive women who had undergone ART during 1996–2007 were invited and altogether 505 women participated in the study.

**Interventions:**

A postal enquiry with a life satisfaction scale.

**Main Outcome Measure:**

Self-reported life satisfaction in respect to the time since the last ART.

**Results:**

In general, women who achieved a live birth after ART had a significantly higher life satisfaction than those who had unsuccessful ART, especially when compared in the first three years. The difference disappeared in the time period of 6–9 years after ART. The unsuccessfully treated women who had a child by some other means before or after the unsuccessful ART had comparable life satisfaction with successfully treated women even earlier.

**Conclusions:**

Even if unsuccessful ART outcome is associated with subsequent lower level of life satisfaction, it does not seem to threaten the long-term wellbeing.

## Introduction

Involuntary childlessness is regarded as a major life crisis. The prevalence of infertility has been estimated to range from 417% [1]. In Finland it has been reported to affect one out of five women of childbearing age and over half of them seem to have sought medical help for it [2]. Nevertheless, about 3050% of couples who underwent assisted reproductive technologies (ART) such as in vitro fertilization (IVF) and intra cytoplasmic sperm injection (ICSI) remain childless [3].

The inability to conceive may cause successive disappointments to the couple [4]. Two out of every three women have been reported to remember the time period of infertility as one of the worst and most stressful experiences in their life [5]. Two comprehensive literature reviews on infertility specific distress stated that infertility is associated with distress, which is reflected by higher levels of depression and anxiety among infertile women than fertile counterparts, but still the overall clinical importance is limited in the spectrum of psychiatric disorders [6]–[7]. Infertility experience is stronger among women than among men and it varies a lot around the world [6]–[7].

In general, IVF/ICSI treatments are loaded with lots of expectations. Even if the couple may try to be realistic in relation to the treatment, optimism is prone to rise during the ART. Often emotions swing from hopefulness and enthusiasm to sorrow and frustration. High levels of anxiety and depression including suicidal thoughts have been reported to be linked with unsuccessful ART [8]–[10]. Furthermore, the risk of hospitalizations for psychiatric reasons is higher among unsuccessfully treated women than among those with successful ART [11]. Involuntary childlessness might also lead to other adverse outcomes such as isolation and social estrangement [12]. Infertile couple may avoid social life with families with children to protect the couple from facing uncomfortable situations. Due to differences in the social stigma caused by infertility in different cultures the degree of isolation may vary substantially [7]. Thus, infertility and failure in ART is a matter of quality of life [13] and life satisfaction [14], [15]. For example, Hammaberg et al. have reported that women with unsuccessful infertility treatments have significantly lower life satisfaction [16]. A study from Norway found that 19% of childless infertile women were dissatisfied with their life compared with 14% of fertile women with a child [17]. However, a systematic review of Verhaak et al. (2007) found that most women adjust well to unsuccessful IVF-treatments even if a significant proportion of women suffer from several adverse consequences [18]. So far there is little evidence on how long it takes to adjust to infertility. For example, it has been shown that three years since an unsuccessful IVF, most women still had the grief process unresolved [9]. Furthermore, women who had had unsuccessful ART 5 years earlier and were childless suffered from lower general wellbeing than women with successful ART outcome [19]. Instead, 10 years after infertility treatments the quality of life seems to be comparable in childless women and those who succeeded in the treatments [20].

Subjective wellbeing - indicated by life satisfaction and happiness - is one of the main dimensions of mental health [21]. It can be affected by achievement of one’s life goals and personal ability to adjust to a major life setback, e.g. involuntary childlessness. Like in any medical treatment, one of the main goals of infertility treatment is not only to improve somatic outcomes, but also to produce subjective wellbeing. In the management of infertility, it is to help the couple to achieve a resolution to the crisis of childlessness, regardless whether a live birth is achieved or not. Thus, support and improvement of adjustment to childlessness are important in counselling.

The present study was undertaken to compare life satisfaction between women with a live birth after ART and women with unsuccessful ART by taking into account the time since the ART.

## Methods

### Ethics Statement

The study was approved by the Ethics Committee of Kuopio University Hospital. Written informed consent was given by participants for their clinical data to be used in the study and data of non-responding women were not analyzed, except live birth rate information was obtained anonymously from The Finnish Medical Birth Register.

### Study participants

A total of 987 consecutive women underwent an in vitro fertilization (IVF) or intra cytoplasmic sperm injection (ICSI) treatment in Kuopio University Hospital between October 1996 and February 2007. All of them were sent a postal questionnaire in June 2008 with an invitation to the study, a written informed consent and an information sheet on opportunities for professional help if needed due to infertility-related problems. In addition to the questionnaire, data on their IVF/ICSI-treatment (details of ART, i.e. number of treatment cycles, transferred fresh and frozen embryos, outcome of treatments, time of the last ART), women’s body mass index and age of the couple were collected from The Fertility Register of the infertility unit in Kuopio University Hospital. The Finnish Medical Birth Register provided a live birth rate of the non-responding women and this information was anonymous.

Altogether, 540 women participated in the study. Consequently, the response rate was 54.7% after one reminder letter. Of these women, 35 had visited private fertility clinics after receiving ART in Kuopio University Hospital. They were excluded due to the inability to obtain detailed information on their treatment in the private sector. This study did not examine the reasons of the clientele to visit private clinics but the most common reasons were that they already have had the maximum number of three publicly funded IVF treatments or that they required a more flexible schedule.

In Finland, ART is offered by both the public and private sectors. ART in public health care is available only to couples who have no more than one common child. The strict age limit is not determined but usually women aged over 40 years are not treated in the public health care, unless the possibility to have success in ART is estimated to be better than 10% (for example sufficient antral follicle count). Principally, the maximum number of IVF treatments is three, and extra stimulation cycles are possible if the next treatment is evaluated to be worthwhile, due to, for instance, miscarriage in the previous cycle. The cost of medications is publicly reimbursed so that a patient has to pay only up to a maximum of about 700€/year.

### The questionnaire

The postal questionnaire included several questions about ART (treatment before and during ART, outcome and cessation), couple’s decisions after ART, psychosocial support and a four-item life satisfaction scale (LS). The latter has a strong association (r = 0.6–0.8) with the 21-item Beck Depression Inventory (BDI) [22], but unlike depression scales it evaluates also the positive pole of mental health, i.e. subjective well-being. Previous studies on LS have proved parallel findings or a statistical significant relationship with other well-established measures such as General Health Questionnaire GHQ-20 and GHQ-12 [23], [24]. LS has been modified from quality of life studies [25]–[27] and used to measure life satisfaction of the general as well as psychiatric populations [28]–[30]. Previous studies have documented its usability to predict long-term mortality, morbidity and health habits in the general population [31]–[35] as well its associations in patient populations with health-related quality of life [36], health habits [24], [37], psychiatric [31] and surgical recovery processes [38] indicated by several well known and widely used scales.

The four items of the LS are following: Do you feel that your life at present is a) very **interesting** (1), fairly interesting (2), fairly boring (4) or very boring (5)? b) very **happy** (1), fairly happy (2), fairly sad (4) or very sad (5)? c) very **easy** (1), fairly easy (2), fairly hard (4) or very hard (5)? d) Do you feel that at the present moment you are very **lonely** (5), fairly lonely (4) or not at all lonely (1)? The scores are presented in the parenthesis. The response “cannot say” or omitting an item is scored as 3. If three or all four questions were omitted, the score was recorded as a missing data. Thus, the total LS score ranges from 4 to 20. As in previous studies the total score was divided to three categories based on the distribution in a general population; the score 4–6 indicated satisfaction, 12–20 dissatisfaction and the intermediate group (LS 7–11) consisted of those subjects within one standard deviation from the mean [25]. All questions were answered by 98.4% and at least two questions by 99.4% of responders. Thus, only three subjects (0.6%) were excluded due to incomplete response to LS.

### Statistical analyses

The comparison of the two groups, i.e. the women who had a live birth as a result of ART and those who had unsuccessful outcome, was performed using SPSS version 17.0 (SPSS Inc., Chicago, IL, USA). When time was taken into consideration, the comparisons were conducted between three groups: i) women with a live birth after ART, ii) women with unsuccessful ART but with a biological child either before or after ART, and iii) childless women with unsuccessful ART. The statistical significance of continuous variables was analysed using a two-tailed pooled t tests, and Mann-Whitney U-test when appropriate, and displayed as mean ± standard deviation (SD). Frequencies were compared by the χ^2^-test or by Fisher’s exact test, as appropriate. A value of p<0.05 was considered statistically significant. The risk ratios for being satisfied (LS 4–6) were calculated by using unadjusted relative risk ratios comparing successfully treated women with unsuccessfully treated women with or without children.

## Results

### Background

The final study group (n = 505) consisted of 296 women with successful and 209 women with unsuccessful ART. Their background data were comparable, with the exception that the latter group was somewhat older and had a slightly higher body mass index (Table 1). The mean age (33.4±5.9 vs. 34.1±5.9) of spouses and their current smoking (28.0% vs. 30.6%) were also similar in these two groups.

**Table 1:** Characteristics of study subjects and the treatments by ART outcome.

Characteristics	Successful ART	Unsuccessful ART	*P*-value
	n = 296	n = 209	
Mean age (years ±SD)	31.0±4.3	31.8±4.8	0.045
BMI (kg/m^2^)	23.6±4.0	24.8±4.9	0.004
Smoking, daily [n (%)]	32 (10.8)	28 (13.4)	NS
Occupation during ART [n (%)]			
Employed	269 (90.9)	192 (91.9)	NS
Unemployed	9 (3.0)	8 (3.8)	NS
Student or other	18 (6.1)	9 (4.3)	NS
Educational level* [n (%)]			
Basic	20 (6.8)	12 (5.7)	NS
Upper secondary	50 (16.9)	32 (15.3)	NS
Tertiary	226 (76.4)	165 (78.9)	NS
Pregnancies before ART [n (%)]	86 (29.1)	73 (34.9)	NS
Biological child before ART [n (%)]	46 (15.5)	39 (18.7)	NS
Indication of ART [n (%)]			
Female factor	115 (38.9)	87 (41.0)	NS
Endometriosis	59 (19.9)	52 (24.9)	NS
Tubal factor	34 (11.5)	17 (8.1)	NS
Uterine factor	0	6 (2.9)	0.003
Anovulation	22 (7.4)	8 (3.8)	NS
PCOS	21 (7.1)	11 (5.3)	NS
Not specified	16 (5.4)	10 (4.8)	NS
Male factor	43 (14.5)	19 (9.1)	NS
Combined factor	37 (12.5)	17 (8.1)	NS
Unexplained	101 (34.2)	86 (41.1)	NS
Duration of infertility (y ± SD)	3.4±2.2	3.5±2.4	NS
Numbers of (mean ± SD)			
Oocyte retrievals	2.1±1.2	2.5±1.3	0.001
Fresh embryo transfers	2.0±1.1	2.4±1.3	0.001
Frozen embryo transfers	1.6±0.9	1.9±1.3	0.045
Miscarriage or ectopic pregnancy [n (%)]	41 (13.9)	59 (28.2)	0.0001
Time from the last ART (y ± SD)	5.6±3.2	5.5±3.4	NS

ART = assisted reproductive technology.

*In the general Finnish women aged 25–44 years, 11.3% had basic education (i.e. Comprehensive school), 39.9% upper secondary education and 48.8% tertiary level education.

Indications for the ART were similar in both ART outcome groups (Table 1). If one includes frozen embryo transfers, 55.0% of women had 1*–*2 transfers, and 40.6% had three or more transfers. The rest of the women had undergone oocyte retrieval without a transfer due to an unsuccessful stimulation. The time since the last ART ranged between 1.3 and 11.7 years (mean: 5.6 y; median: 5.2 y).

Women with unsuccessful ART had had significantly more miscarriages, ectopic pregnancies and ART trials than those with successful ART (Table 1). Duration of infertility, time since last ART and indications for the ART were similar in these two ART outcome groups (Table 1). However, no cases with uterine factor as an indication for ART were found among women with successful ART compared to six cases among women with unsuccessful ART.

In the successful ART group 46 women had a biological child before the treatment. In the unsuccessful ART group, as many as 78 women (37.3%; 78/209) had a biological child either before (n = 39, 18.7%) or after the treatment (n = 47, 22.5%) with altogether eight of women having a child both before and after an unsuccessful treatment.

### ART outcome and life satisfaction

Women with successful ART were significantly more satisfied with their lives in 2008 than the women with unsuccessful ART (mean LS 6.8±2.4 vs. 7.6±3.0, p = 0.003), this was also seen in the proportion of dissatisfied women in these two groups (Table 2). In the intermediate category, instead, there were no differences between the groups. When women with unsuccessful treatment were categorized into two groups according their motherhood status (without a child or with a child without ART either before or after unsuccessful ART treatment), there were no differences when the latter group was compared to the women with successful ART.

**Table 2:** Life satisfaction by ART outcome and offspring status.

	Successful ART	Unsuccessful ART					
	ALL	ALL	*P*-value	CHILD+	*P*-value	CHILD–	*P*-value
	n = 296	n = 209		n = 78		n = 131	
**Life satisfaction score**							
(range 4–20)							
mean LS ± SD	6.8±2.4	7.6±3.0	0.003	7.3±2.9	NS	7.7±3.1	0.003
**Satisfied**, n (%)	154 (52.0)	91 (43.5)	NS	37 (47.4)	NS	54 (41.2)	0.04
(score 4–6)							
**Intermediate**, n (%)	125 (42.2)	92 (44.0)	NS	34 (43.6)	NS	58 (44.3)	NS
(score 7–11)							
**Dissatisfied**, n (%)	17 (5.7)	26 (12.4)	0.008	7 (9.0)	NS	19 (14.5)	0.003
(score 12–20)							

ART = assisted reproductive technology.

CHILD +/− = women with/without a biological child.

Successfully treated women displayed as a control group.

**Figure 1:**
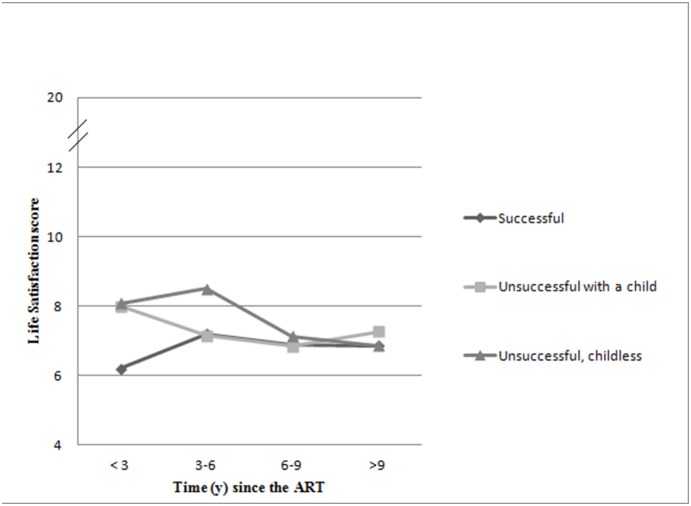
The association of time since the last ART with life satisfaction. The score 4–6 indicates satisfaction, 1220 dissatisfaction and the intermediate group (LS 7–11) consisted of those subjects within one standard deviation from the mean.

When the time since last ART was taken into account, the relationship between LS-score and outcome of ART displayed more variation (Table 3 and Fig. 1). If less than three years have gone after the ART, women with unsuccessful ART with or without a biological child had similar LS scores. Both of these groups were statistically significantly more dissatisfied than successfully treated women. However, if 3*–*6 years had elapsed since the last treatment, only the childless women with unsuccessful ART were still more dissatisfied compared to successfully treated women. If over 6 years has gone after ART, no statistically significant differences in life satisfaction were found between any of these three groups.

**Table 3:** Life Satisfaction score (mean ±SD) by time since last previous ART and risk ratios in respect to life satisfaction between successfully treated women with unsuccessfully treated women with (Child+) or without (Child-) a biological child.

Women with	Time since the last ART				
	<3 years	3–6 years	6–9 years	>9 years	All
**Successful ART**					
n	82	86	75	53	296
mean ±SD	6.2±2.0^a^^,^ b	7.2±2.4^b^	6.9±2.5	6.9±2.6	6.8±2.4^b^
RR, 95% CI	1	1	1	1	1
**Unsuccessful ART, Child+**					
n	17	22	24	15	78
mean ±SD	8.0±3.4	7.1±2.3	6.8±3.0	7.3±3.2	7.3±2.9
RR, 95% CI	0.6 (0.3–1.1)	1.1 (0.6–1.8)	1.2 (0.8–1.8)	1.0 (0.6–1.7)	0.9 (0.7–1.2)
**Unsuccessful ART, Child–**					
n	50	26	26	29	131
mean ±SD	8.1±3.3	8.5±2.9	7.12±2.6	6.9±3.0	7.7±3.1
RR, 95% CI	0.6 (0.4–0.9)	0.5 (0.3–1.1)	0.8 (0.5–1.4)	1.1 (0.8–1.7)	0.8 (0.6–1.0)

ART = assisted reproductive technology; RR = Risk ratio; CI = Confidence interval.

CHILD +/− = women with/without a biological child.

a Statistically significant different (*P-value*<0.05), the comparison between successfully treated women with unsuccessfully treated women who had a biological child.

b Statistically significant different (*P-value*<0.05), the comparison between successfully treated women with unsuccessfully treated women who remained childless.

According to The Finnish Medical Birth Register the live birth rate of the non-responding women (n = 447) was comparable with that of our final study group (n = 540) i.e. 57.5% vs. 58.6%. The mean age of the non-responding women was higher than in the responding women (40.1 *vs*. 38.8 y).

## Discussion

The present study investigated how the outcome of ART is associated with women’s life satisfaction and how time since last ART is relevant to it. Women who had given birth through ART had higher life satisfaction than the women who did not conceive, especially if the time lag was less than three years. However, passing of time (i.e. more than 6 years) and having a biological child by some other means before or after ART seemed to buffer between unsuccessful ART and life satisfaction.

There is little evidence on how the duration of time from the last ART is associated with life satisfaction. Previously, Leiblum et al. (1998) found no association between time since the last treatment (2 to 13 years ago) and life satisfaction in their cross-sectional study [15]. Still, findings supporting our results can be found. According to Verhaak et al. (2005) adjustment to unsuccessful IVF did not occur after six months from IVF in over 20% of their patients [39]. Hammarberg et al. (2001) reported that lower life satisfaction was found after 2–3 years of treatment cessation among women who failed to conceive than among women who succeeded [40]. In a Swedish study, after 5 years since ART, a group with successful IVF had significantly higher general wellbeing than participants in unsuccessful IVF group [19]. Recently, Wischmann (2012) found that three out of four childless women had no further desire for a child after ten years from the first treatment contact [20]. When combining these findings with ours, the adjustment to involuntary childlessness may take place by five to ten years, at least in the majority of the women. Nevertheless, this result does not take into account that some women may have had psychological support to relieve the grief of childlessness.

The present study found positive messages. Firstly, great majority of women with previous ART were satisfied with their life. Only 5.7% of the successfully treated women and 14.5% of women who remained childless were subsequently dissatisfied according the LS scale. The group difference was significant also in the category of satisfied women, but in the intermediate group the proportions of women in the successful and unsuccessful group were equal. The categories of satisfied and unsatisfied represent responses’ extremities while in the intermediate group other factors in life may have diluted the responses. The mean LS score among women undergoing ART was lower (i.e. indicating better life satisfaction) in adult women aged somewhat the same, but the latter data was collected decades ago [30] without taking into account marital status.

In a previous study, the level of psychosocial stress among involuntarily childless women was in the normal range, even if it was higher than in women with successful infertility treatments [41]. Selection may partly explain this. Our participants had higher educational level than women of the same age group in the general Finnish population which may reflect better life management and consequently better adjustment to the possibility of infertility [42]. Moreover, couples willing to attend ART treatment might have better life management than childless couples not capable or willing to the stressful fertility treatment [4]. Furthermore, in a recent Danish study, women who attended ART had lower rate of depression diagnosis than the age-matched general population and the risk of depression decreased within few years after ART [43]. On the one hand, women who do not participate to infertility treatments might have lower desire for a child [44]. Still, both life dissatisfaction [22] as well as unsuccessful IVF has been repeatedly associated with depression [2], [14], [45]–[47]. Recurrent failures in ART can increase the risk of depression, but also miscarriages or ectopic pregnancies, which are more common among women with unsuccessful ART, play a role. This should be taken into consideration in counseling infertile couples, especially at the time of decisions after unsuccessful attempts.

The second positive message was that women with unsuccessful ART were, in general, adjusted to their infertility after few years. However, further longitudinal follow-up studies are needed, especially those including also childless women who do not attempt ART at all.

Overall, one strength in this cross sectional study was the comparable background data of both groups, i.e. those with successful or unsuccessful outcome as for educational level, occupation during ART, motherhood status and duration of infertility. We used the valid self-assessment scales and register based information of the actual time since last ART was reliable. The life satisfaction scale we have chosen has a strong correlation with Beck Depression Inventory (BDI) [22], which is a widely used instrument for depression. BDI, however, does not measure positive aspects of life in contrast to the life satisfaction scale. Our survey focused on women who were public health care clients. Thus, access to ART and treatment procedures was equal for all, regardless of the social status. All women were treated in the same clinic by the same physicians and were living in the same district.

Despite the strengths mentioned above this study has some limitations, such as a relatively low response rate (55%) but it is in line with other infertility related surveys [40]. According to non-responder analysis, the non-responders were statistically significantly somewhat older (1.3years) than women in the study groups. However, its clinical significance was unlikely crucial in this setting. One can speculate also that women with advancing age may have somewhat diluted significance of infertility. Live birth rate, on the contrary, was equal among the responders and non-responders (59% vs. 58%, respectively). Still, women with decreased ability to process their infertility, might respond less willingly to a questionnaire about infertility. If they had responded, it might have increased the life satisfaction differences between ART outcome groups. Thus, our results may be a conservative.

In the present study the group of women who did not consider ART as a possible treatment option could not be assessed. Only about every second woman with infertility will seek help and two thirds of them have been reported to discontinue fertility care before attempting IVF [48]. A limitation was also that the spouses were not included, even if infertility affects also men or couples as a whole. Still, according to previous studies, infertility has a greater emotional effect on women than on men [15], [49]–[51].

In a cross sectional study design it is not possible to show causalities. Thus, we do not claim that the success in ART will cause higher life satisfaction, but we were able to point the association between ART outcome, life satisfaction and time passing. Still, we did not have potential to take into account other life occurrences such as adoption, divorce or severe illness. Previously, it has been shown that mothers through adoption have higher general wellbeing than women with non-successful IVF or mothers through spontaneous pregnancy [19]. In a longitudinal setting there is a problem of declining response rate over time and selection bias in the study subjects. In our study, the distribution of the responders was equal at the different time points. They also filled in the questionnaire at the same time point. Thus, environmental factors such as economic highs and lows in the society did not affect the responses in the present study.

This study was conducted in Finland, where it is, like in other developed countries, socially acceptable to be voluntarily childless. In low-income settings, on the contrary, involuntary childless women may feel themselves as a society’s outcast [52]. By taking into consideration these circumstances, including that in Finland ART is available for all social classes, the results of the current study can be generalized to women living in developed countries with a similar structure of the society and to women who have a chance to attend ART.

In conclusion, even if infertility is a source of grief and a risk for poor well-being it can be a life crisis which seems not to have a tendency to jeopardize the long-term well-being of a woman in developed countries. Nevertheless, the current study does not allege that the grief will disappear, but it is possible to learn, after obtaining help from professionals or family if necessary, to live alongside with it.

## References

[1] Boivin J, Bunting L, Collins JA, Nygren KG (2007) International estimates of infertility prevalence and treatment-seeking: Potential need and demand for infertility medical care. Hum Reprod 22: 1506–1512. 17376819 10.1093/humrep/dem046

[2] Klemetti R, Raitanen J, Sihvo S, Saarni S, Koponen P (2010) Infertility, mental disorders and well-being–a nationwide survey. Acta Obstet Gynecol Scand 89: 677–682. 20196679 10.3109/00016341003623746

[3] Malizia BA, Hacker MR, Penzias AS (2009) Cumulative live-birth rates after in vitro fertilization. N Engl J Med 360: 236–243. 19144939 10.1056/NEJMoa0803072

[4] Cousineau TM, Domar AD (2007) Psychological impact of infertility. Best Pract Res Clin Obstet Gynaecol 21: 293–308. 17241818 10.1016/j.bpobgyn.2006.12.003

[5] Sundby J, Schmidt L, Heldaas K, Bugge S, Tanbo T (2007) Consequences of IVF among women: 10 years post-treatment. J Psychosom Obstet Gynaecol 28: 115–120. 17538819 10.1080/01674820701447447

[6] Greil AL (1997) Infertility and psychological distress: A critical review of the literature. Soc Sci Med 45: 1679–1704. 9428088 10.1016/s0277-9536(97)00102-0

[7] Greil AL, Slauson-Blevins K, McQuillan J (2010) The experience of infertility: A review of recent literature. Sociol Health Illn 32: 140–162. 20003036 10.1111/j.1467-9566.2009.01213.x PMC3383794

[8] Verhaak CM, Smeenk JM, Nahuis MJ, Kremer JA, Braat DD (2007) Long-term psychological adjustment to IVF/ICSI treatment in women. Hum Reprod 22: 305–308. 16973721 10.1093/humrep/del355

[9] Volgsten H, Svanberg AS, Olsson P (2010) Unresolved grief in women and men in sweden three years after undergoing unsuccessful in vitro fertilization treatment. Acta Obstet Gynecol Scand 89: 1290–1297. 20846062 10.3109/00016349.2010.512063 PMC2993044

[10] Volgsten H, Skoog Svanberg A, Ekselius L, Lundkvist O, Sundstrom Poromaa I (2010) Risk factors for psychiatric disorders in infertile women and men undergoing in vitro fertilization treatment. Fertil Steril 93: 1088–1096. 19118826 10.1016/j.fertnstert.2008.11.008

[11] Yli-Kuha AN, Gissler M, Klemetti R, Luoto R, Koivisto E, et al (2010) Psychiatric disorders leading to hospitalization before and after infertility treatments. Hum Reprod 25: 2018–2023. 20570970 10.1093/humrep/deq164

[12] Schmidt L, Holstein BE, Christensen U, Boivin J (2005) Communication and coping as predictors of fertility problem stress: Cohort study of 816 participants who did not achieve a delivery after 12 months of fertility treatment. Hum Reprod 20: 3248–3256. 16006458 10.1093/humrep/dei193

[13] Chachamovich JR, Chachamovich E, Ezer H, Fleck MP, Knauth D, et al (2010) Investigating quality of life and health-related quality of life in infertility: A systematic review. J Psychosom Obstet Gynaecol 31: 101–110. 20443659 10.3109/0167482X.2010.481337

[14] Weaver SM, Clifford E, Hay DM, Robinson J (1997) Psychosocial adjustment to unsuccessful IVF and GIFT treatment. Patient Educ Couns 31: 7–18. 9197798 10.1016/s0738-3991(97)01005-7

[15] Leiblum SR, Aviv A, Hamer R (1998) Life after infertility treatment: A long-term investigation of marital and sexual function. Hum Reprod 13: 3569–3574. 9886552 10.1093/humrep/13.12.3569

[16] Hammarberg K, Astbury J, Baker H (2001) Women's experience of IVF: A follow-up study. Hum Reprod 16: 374–383. 11157838 10.1093/humrep/16.2.374

[17] Rostad B, Schmidt L, Sundby J, Schei B (2014) Infertility experience and health differentials - a population-based comparative study on infertile and non-infertile women (the HUNT study). Acta Obstet Gynecol Scand 93: 757–764. 24773205 10.1111/aogs.12404

[18] Verhaak CM, Smeenk JM, Evers AW, Kremer JA, Kraaimaat FW, et al (2007) Women's emotional adjustment to IVF: A systematic review of 25 years of research. Hum Reprod Update 13: 27–36. 16940360 10.1093/humupd/dml040

[19] Högstrom L, Johansson M, Janson PO, Berg M, Francis J, et al (2012) Quality of life after adopting compared with childbirth with or without assisted reproduction. Acta Obstet Gynecol Scand 91: 1077–1085. 22708621 10.1111/j.1600-0412.2012.01491.x

[20] Wischmann T, Korge K, Scherg H, Strowitzki T, Verres R (2012) A 10-year follow-up study of psychosocial factors affecting couples after infertility treatment. Hum Reprod 27: 3226–3232. 22888171 10.1093/humrep/des293

[21] Vaillant GE (2003) Mental health. Am J Psychiatry 160: 1373–1384. 12900295 10.1176/appi.ajp.160.8.1373

[22] Koivumaa-Honkanen H, Kaprio J, Honkanen R, Viinamäki H, Koskenvuo M (2004) Life satisfaction and depression in a 15-year follow-up of healthy adults. Soc Psychiatry Psychiatr Epidemiol 39: 994–999. 15583908 10.1007/s00127-004-0833-6

[23] Linna MS, Kaprio J, Raevuori A, Sihvola E, Keski-Rahkonen A, et al (2013) Body mass index and subjective well-being in young adults: A twin population study. BMC Public Health 13: 231–2458-13-231. 23496885 10.1186/1471-2458-13-231 PMC3691623

[24] Rissanen T, Viinamäki H, Honkalampi K, Lehto SM, Hintikka J, et al (2011) Long term life dissatisfaction and subsequent major depressive disorder and poor mental health. BMC Psychiatry 11: 140–244X-11-140. 21861908 10.1186/1471-244X-11-140 PMC3170593

[25] Allardt E (1973) About dimension of welfare: An explanatory analysis of the comparative scandinavian survey. University of Helsinki reseach group of comparative sociology reseach report 1. Helsinki, Finland; University of Helsinki.

[26] Andrews F, Withey S (1976) Social indicators of well-being: Americans’ perception of life quality. New York: Plenum Press.

[27] Campbell A, Converse P, Rodgers W (1976) The quality of American life. New York: Russell Stage Foundation.

[28] Koivumaa-Honkanen HT, Viinamäki H, Honkanen R, Tanskanen A, Antikainen R, et al (1996) Correlates of life satisfaction among psychiatric patients. Acta Psychiatr Scand 94: 372–378. 9124086 10.1111/j.1600-0447.1996.tb09875.x

[29] Koivumaa-Honkanen H, Honkanen R, Antikainen R, Hintikka J, Laukkanen E, et al (2001) Self-reported life satisfaction and recovery from depression in a 1-year prospective study. Acta Psychiatr Scand 103: 38–44. 11202127 10.1034/j.1600-0447.2001.00046.x

[30] Koivumaa-Honkanen H, Kaprio J, Honkanen RJ, Viinamäki H, Koskenvuo M (2005) The stability of life satisfaction in a 15-year follow-up of adult finns healthy at baseline. BMC Psychiatry 5: 4. 15656900 10.1186/1471-244X-5-4 PMC546219

[31] Koivumaa-Honkanen H, Honkanen R, Viinamäki H, Heikkila K, Kaprio J, et al (2000) Self-reported life satisfaction and 20-year mortality in healthy finnish adults. Am J Epidemiol 152: 983–991. 11092440 10.1093/aje/152.10.983

[32] Koivumaa-Honkanen H, Honkanen R, Viinamäki H, Heikkilä K, Kaprio J, et al (2001) Life satisfaction and suicide: A 20-year follow-up study. Am J Psychiatry 158: 433–439. 11229985 10.1176/appi.ajp.158.3.433

[33] Koivumaa-Honkanen H, Honkanen R, Koskenvuo M, Viinamäki H, Kaprio J (2002) Life dissatisfaction as a predictor of fatal injury in a 20-year follow-up. Acta Psychiatr Scand 105: 444–450. 12059849 10.1034/j.1600-0447.2002.01287.x

[34] Koivumaa-Honkanen H, Koskenvuo M, Honkanen RJ, Viinamäki H, Heikkilä K, et al (2004) Life dissatisfaction and subsequent work disability in an 11-year follow-up. Psychol Med 34: 221–228. 14982128 10.1017/s0033291703001089

[35] Koivumaa-Honkanen H, Kaprio J, Korhonen T, Honkanen RJ, Heikkilä K, et al (2012) Self-reported life satisfaction and alcohol use: A 15-year follow-up of healthy adult twins. Alcohol Alcohol 47: 160–168. 22215005 10.1093/alcalc/agr151

[36] Saharinen T, Koivumaa-Honkanen H, Hintikka J, Kylmä J, Lehto SM, et al. (2013) The effect of long-term life dissatisfaction on health-related quality of life among general population subjects. J Psychiatr Ment Health Nurs. 10.1111/jpm.12060 23527583

[37] Koivumaa-Honkanen H, Kaprio J, Korhonen T, Honkanen RJ, Heikkilä K, et al (2012) Self-reported life satisfaction and alcohol use: A 15-year follow-up of healthy adult twins. Alcohol Alcohol 47: 160–168. 22215005 10.1093/alcalc/agr151

[38] Pakarinen M, Koivumaa-Honkanen H, Sinikallio S, Lehto SM, Aalto T, et al (2014) Life dissatisfaction burden is associated with a poor surgical outcome among lumbar spinal stenosis patients: A 5-year follow-up study. Int J Rehabil Res 37: 80–85. 24135635 10.1097/MRR.0000000000000039

[39] Verhaak CM, Smeenk JM, van Minnen A, Kremer JA, Kraaimaat FW (2005) A longitudinal, prospective study on emotional adjustment before, during and after consecutive fertility treatment cycles. Hum Reprod 20: 2253–2260. 15817584 10.1093/humrep/dei015

[40] Hammarberg K, Astbury J, Baker H (2001) Women's experience of IVF: A follow-up study. Hum Reprod 16: 374–383. 11157838 10.1093/humrep/16.2.374

[41] Bryson CA, Sykes DH, Traub AI (2000) In vitro fertilization: A long-term follow-up after treatment failure. Hum Fertil (Camb) 3: 214–220. 11844381 10.1080/1464727002000199011

[42] Educational structure of population. Helsinki: Official Statistics of Finland (OSF); 2010. Available: http://www.stat.fi/til/vkour/index_en.html. Accessed 2010 Dec 3.

[43] Sejbaek CS, Hageman I, Pinborg A, Hougaard CO, Schmidt L (2013) Incidence of depression and influence of depression on the number of treatment cycles and births in a national cohort of 42,880 women treated with ART. Hum Reprod 28: 1100–1109. 23300199 10.1093/humrep/des442

[44] Greil AL, Shreffler KM, Schmidt L, McQuillan J (2011) Variation in distress among women with infertility: Evidence from a population-based sample. Hum Reprod 26: 2101–2112. 21659313 10.1093/humrep/der148 PMC3137388

[45] Slade P, Emery J, Lieberman BA (1997) A prospective, longitudinal study of emotions and relationships in in-vitro fertilization treatment. Hum Reprod 12: 183–190. 10.1093/humrep/12.1.183 9043926

[46] Kee BS, Jung BJ, Lee SH (2000) A study on psychological strain in IVF patients. J Assist Reprod Genet 17: 445–448. 11062855 10.1023/A:1009417302758 PMC3455575

[47] Johansson M, Adolfsson A, Berg M, Francis J, Hogstrom L, et al (2010) Gender perspective on quality of life, comparisons between groups 4–5.5 years after unsuccessful or successful IVF treatment. Acta Obstet Gynecol Scand 89: 683–691. 20302532 10.3109/00016341003657892

[48] Brandes M, van der Steen JO, Bokdam SB, Hamilton CJ, de Bruin JP, et al (2009) When and why do subfertile couples discontinue their fertility care? A longitudinal cohort study in a secondary care subfertility population. Hum Reprod 24: 3127–3135. 19783833 10.1093/humrep/dep340

[49] Hjelmstedt A, Andersson L, Skoog-Svanberg A, Bergh T, Boivin J, et al (1999) Gender differences in psychological reactions to infertility among couples seeking IVF- and ICSI-treatment. Acta Obstet Gynecol Scand 78: 42–48. 9926891

[50] Newton CR, Sherrard W, Glavac I (1999) The fertility problem inventory: Measuring perceived infertility-related stress. Fertil Steril 72: 54–62. 10428148 10.1016/s0015-0282(99)00164-8

[51] Wichman CL, Ehlers SL, Wichman SE, Weaver AL, Coddington C (2011) Comparison of multiple psychological distress measures between men and women preparing for in vitro fertilization. Fertil Steril 95: 717–721. 21067728 10.1016/j.fertnstert.2010.09.043

[52] Cui W (2010) Mother or nothing: The agony of infertility. Bull World Health Organ 88: 881–882. 21124709 10.2471/BLT.10.011210 PMC2995184

